# Targeted gene sequencing and bioinformatics analysis of a patient with gallbladder adenosquamous carcinoma: a case report

**DOI:** 10.3389/fonc.2026.1697015

**Published:** 2026-01-26

**Authors:** Yangjun Gu, Zhitao Chen, Qingqing Fang, Qiyong Li

**Affiliations:** 1 Department of Hepatobiliary and Pancreatic Surgery, Shulan Hangzhou Hospital Affiliated to Zhejiang Shuren University Shulan International Medical College, Hangzhou, China; 2 Department of Plastic Surgery, Zhejiang University School of Medicine Sir Run Run Shaw Hospital, Hangzhou, China

**Keywords:** bioinformatics analysis, case report, combined treatment, gallbladder adenosquamous carcinoma, targeted gene sequencing

## Abstract

**Introduction:**

Gallbladder adenosquamous carcinoma (GBASC) is an uncommon, highly aggressive neoplasm characterized by the coexistence of both glandular and squamous cells. Representing fewer than 5% of gallbladder malignancies, GBASC demonstrates a more aggressive behavior and has poorer prognosis, posing considerable challenges for early diagnosis and effective management.

**Case presentation:**

We present a case of GBASC in a 52-year-old woman who achieved long-term tumor-free survival by surgery, as well as targeted and immunotherapy after the operation. Targeted gene sequencing and bioinformatics analysis tools, including STRING, GeneMANIA, Metascape, TRRUST, Sangerbox, and cBioPortal, were used to analyze the biological functions and features of the mutated genes in GBASC. A total of 16 mutations (*NF2*, *EGFR*, *EPHA2*, *CDK6*, *LATS2*, *NBN*, *CUL3*, *FRAS1*, *ATM*, *KMT2A*, *EXT1*, *SMARCA1*, *RECQL4*, *KMT2D*, *POLQ*, and *CTNND2*) were identified, and the tumor mutation burden was determined to be 5.73 mut/Mb via targeted gene sequencing. The protein–protein interaction network highlighted robust connections among the 16 mutated genes. Functional enrichment via Gene Ontology and Kyoto Encyclopedia of Genes and Genomes pathway analyses pinpointed tumor-associated signaling cascades. Moreover, based on bioinformatics analysis, the key points at the treatment duration of this patient were discussed.

**Conclusions:**

Comparative analyses with other gallbladder carcinoma subtypes revealed GBASC to have distinct clinical phenotypes, molecular alterations, functional characteristics, and enriched signaling pathways. Moreover, there is an urgent need for standardized treatment protocols.

## Introduction

Gallbladder adenosquamous carcinoma (GBASC) is an uncommon, highly aggressive malignancy accounting for fewer than 5% of gallbladder cancers (GBCs). Characterized by the coexistence of glandular and squamous components within the same neoplasm, GBASC exhibits a more aggressive behavior and has poorer prognosis than conventional gallbladder adenocarcinoma (1, 2). Due to the nonspecific nature of the early symptoms, many cases are discovered incidentally at the time of cholecystectomy or after the development of locally advanced or metastatic conditions (3). Risk factors are the same as those of conventional gallbladder adenocarcinoma, including chronic cholelithiasis, porcelain gallbladder, and gallbladder polyps. However, the mechanisms that drive squamous differentiation in a glandular milieu remain poorly understood. Furthermore, the mutational landscape of GBASC, including its key regulatory factors, gene–gene interactions, and enriched signaling pathways, remains poorly defined.

We report on a case of GBASC in a 52-year-old woman, integrating comprehensive histopathological assessment with targeted gene sequencing. This study aimed to characterize the clinical presentation, the molecular alterations, and the management strategies for GBASC and contrasted its biological and genomic features with those of conventional gallbladder adenocarcinoma. The manuscript was prepared in accordance with the CARE checklist to ensure transparent and standardized clinical case reporting.

## Case presentation

A 52-year-old woman was admitted to our department for “epigastric distension over 6 months and worsen in the past month.” The patient developed epigastric distension over 6 months ago without identifiable precipitating factors. Furthermore, there was absence of belching, vomiting, melena, or hematochezia. An upper endoscopy was performed at a local hospital, which revealed reflux esophagitis, non-atrophic gastritis with antral erosions, and bile reflux. Treatment with Chinese herbal medicine, acid suppression, and gastric mucosal protection yielded suboptimal response. In the past month, the abdominal distension progressed and significantly affected sleep. She lost 6 kg in the past month, without edema, anemia, jaundice, hepatomegaly, or splenomegaly. A history of type 2 diabetes was noted. Laboratory examination indicated an elevated white blood cell count and C-reactive protein, slight liver function impairment, and increased carbohydrate antigen 125, but normal carbohydrate antigen 19-9 (CA 19-9).

**Figure 1:**
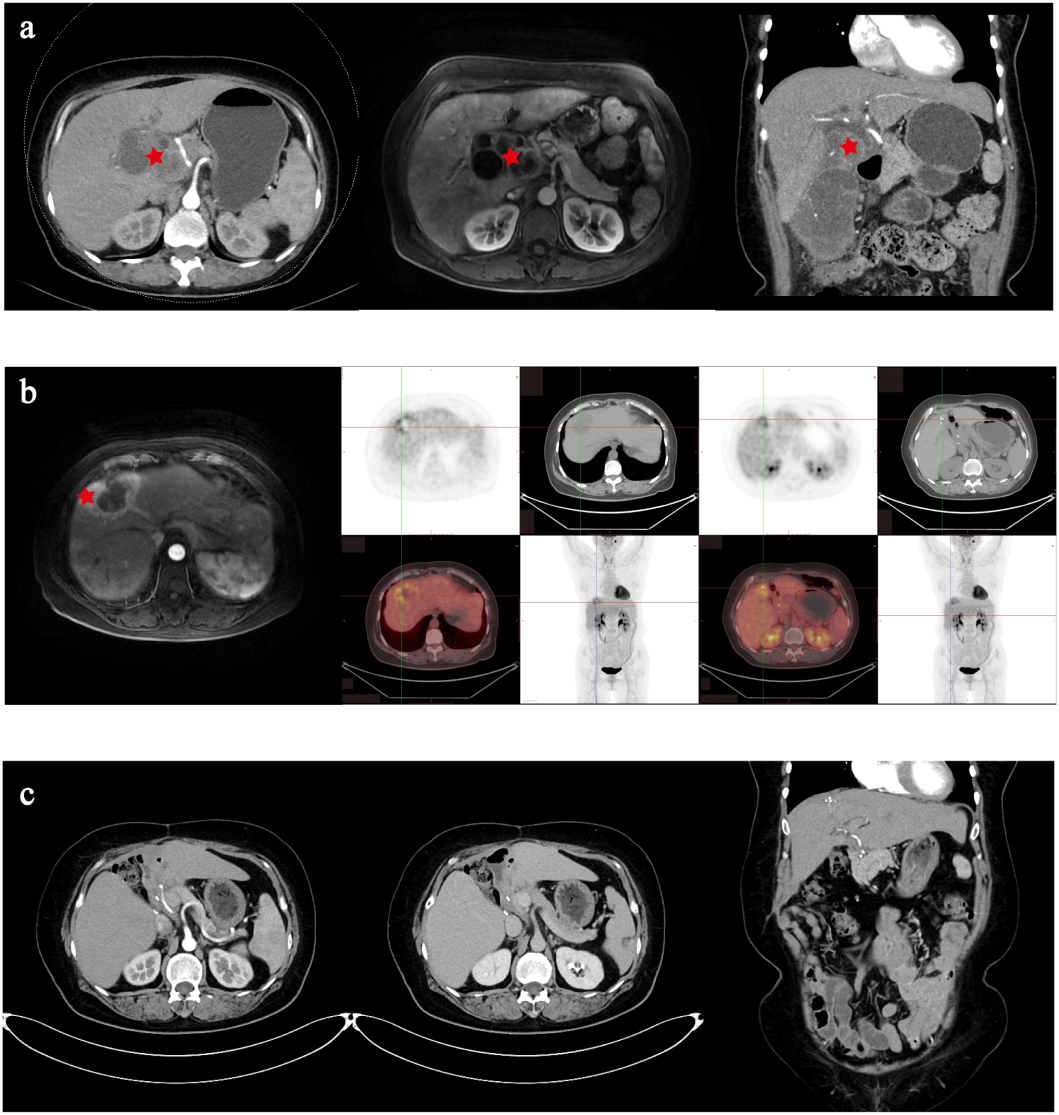
Imaging examination of the patient. **(a)** Contrast-enhanced computed tomography (CT) and contrast-enhanced magnetic resonance indicating locally advanced gallbladder carcinoma. **(b)** Positron emission tomography–CT indicating tumor recurrence in the surgical area. **(c)** Contrast-enhanced CT indicating tumor-free condition at the recent follow-up.

Contrast-enhanced computed tomography (CT) and contrast-enhanced magnetic resonance (MR) showed irregular thickening of the gallbladder wall associated with a soft tissue mass. The giant mass invaded adjacent hepatic parenchyma and biliary ducts, resulting in intrahepatic biliary dilatation. Significant vascular encasement was observed, which involved the common hepatic artery, proper hepatic artery, proximal segments of the right/left hepatic arteries, cystic artery, main portal vein, and its right/left branches (**Figure 1A**). Subsequently, a puncture biopsy was performed, which demonstrated a poorly differentiated carcinoma. After discussion with a multidisciplinary team, she was diagnosed with gallbladder carcinoma with liver invasion and cholelithiasis. On May 4, 2020, extended radical surgical treatment was performed, including radical cholecystectomy, hepatic hilar lymphadenectomy, T-tube drainage, and resection of liver segment IV-B. The postoperative biopsy and immunohistochemical analysis revealed a poorly differentiated adenocarcinoma with extensive necrosis (**Supplementary Figure S1A**), which was positive in periodic acid–Schiff (PAS), CA 19-9, CK19, CK7, MLH1/2/6, P53, and PMS2, with KI-67 of 60%.

Postoperative adjuvant therapy, including gemcitabine [1,000 mg/m^2^, day 1 (D1) and D14] combined with oral tegafur (40 mg BID, D2–D8) and sintilimab (200 mg, D2) was started 6 weeks after surgery. However, the tumor recurred around 5 months after the operation. Subsequently, ultrasound-guided radiofrequency was performed on November 10, 2020 for liver metastatic lesions and the chemotherapy switched to gemcitabine (1,000 mg/m^2^, D1 and D14) and oxaliplatin (80 mg/m^2^, D1 and D14). Unfortunately, 2 months later, the tumors were found to have progressed near the surgical area (**Figure 1B**). After discussion with a multidisciplinary team, partial hepatectomy and abdominal metastasis tumor resection were performed on February 4, 2021. The postoperative biopsy and immunohistochemical analysis revealed a poorly differentiated adenosquamous carcinoma (**Supplementary Figure S1B**), which was positive in CK19, CK7, MLH1/2/6, MOC31, P53, PMS2, P40, and Vim, with KI-67 of 70% and programmed cell death protein 1 (PD-1) of more than 50%. Due to refusal of chemotherapy, the patient received anlotinib (10 mg, QD) and camrelizumab (200 mg, Q3D) at 4 weeks after surgery. Until the recent oncology assessment on July 13, 2025, she achieved radiologic tumor-free survival. The treatment process is shown in **Supplementary Figure S2**.

Comprehensive genetic testing was performed on the tumor DNA extracted from the cancer, encompassing selected introns of 688 cancer-related genes, 15 microsatellite-related genes, immunotherapy-related genes, and tumor mutation burden (TMB) (produced by BGI Genomics Co., Ltd., MGISEQ-2000 platform).

## Discussion

GBASC comprises roughly 5% of the GBCs that typically presents at an advanced stage with nonspecific symptoms such as persistent right upper quadrant pain, jaundice, weight loss, anorexia, and fatigue (1). The imaging manifestations (ultrasound, contrast-enhanced CT, or MRI) of GBASC are similar to those of ordinary GBCs, which characteristically present as an irregular, heterogeneously enhancing mass with wall thickening and early hepatic invasion or nodal involvement. Definitive diagnosis requires histopathology demonstrating both glandular and squamous components (the latter ≥25% of the tumor), and the immunohistochemical profiles could also help define the subtype and predict the prognosis of the disease (4). The outcomes of GBASC have been disappointing, with a median survival of approximately 3.3–3.9 months, while patients with R0 resection have shown longer survival close to that of ordinary GBCs (3, 5). The optimal treatment for GBASC relies on its early diagnosis and radical surgical resection. Due to its high malignancy and early lymphatic metastasis, adjuvant chemotherapy and external beam radiotherapy are recommended to reduce recurrence and prolong survival (5). For unresectable, metastatic, or recurrent presentations, multimodality treatment is essential to individualize the treatment plans, optimize outcomes, and explore novel approaches.

In this case, the pathology of neoplasia changed from adenocarcinoma to adenosquamous carcinoma, which has a more aggressive presentation and a much worse prognosis (6). Many studies have confirmed that cancer treatment imposes strong selective pressures on the tumor cell populations, driving a variety of non-genetic and genetic adaptations that enable them to evade treatments and relapse via phenotypic switching (7). This study reviewed the existing literature and used bioinformatics analysis to compare the clinicopathologic and genetic characteristics of GBASC and conventional gallbladder adenocarcinoma.

**Figure 2:**
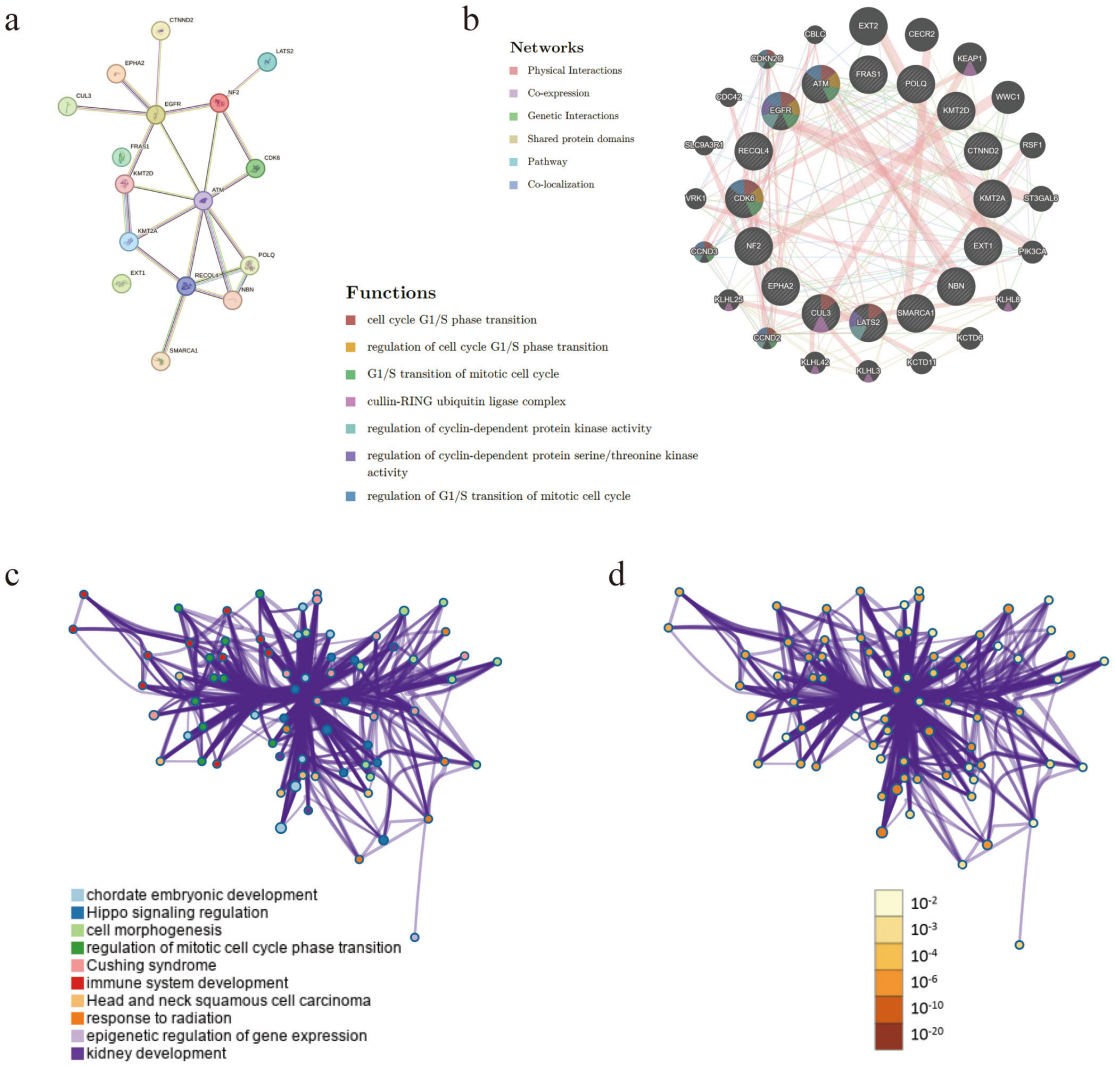
Interaction network analyses. **(a, b)** Protein–protein interaction network of the 16 mutated genes. **(c, d)** Protein–protein interaction enrichment analysis between the 16 mutated genes.

Targeted gene sequencing helped identify 16 mutations (*NF2*, *EGFR*, *EPHA2*, *CDK6*, *LATS2*, *NBN*, *CUL3*, *FRAS1*, *ATM*, *KMT2A*, *EXT1*, *SMARCA1*, *RECQL4*, *KMT2D*, *POLQ*, and *CTNND2*) unique to the specimen (**Table 1**), and the TMB was determined as 5.73 mut/Mb (**Supplementary Figure S3**). To elucidate the biology and to uncover potential therapeutic targets of this rare tumor, we leveraged multiple bioinformatics resources to dissect its oncogenic underpinnings. Firstly, we constructed and visualized a protein–protein interaction (PPI) network in STRING (8) (version 11.5; https://string-db.org/), which comprised 16 nodes and 21 edges, exhibited a high average local clustering coefficient of 0.655, and showed significant PPI enrichment (*p* < 0.0001) (**Figure 2A**). Thereafter, GeneMANIA (9) (http://genemania.org/) analysis highlighted that the mutated genes converge on complexes and processes such as the cell cycle G1/S phase transition, regulation of cell cycle G1/S phase transition, G1/S transition of mitotic cell cycle, cullin–RING ubiquitin ligase complex, regulation of cyclin-dependent protein kinase activity, regulation of cyclin-dependent protein serine/threonine kinase activity, and regulation of G1/S transition of the mitotic cell cycle (**Figure 2B**). Finally, complementary PPI enrichment via Metascape (https://metascape.org/) further validated these interactions and functional clusters (**Figures 2C, D**).

**Table 1:** Information on gallbladder adenosquamous carcinoma (GBASC)-related mutated genes.

Gene	Original name	Cytoband	Variant type	Abundance variation
*NF2*	Moesin–ezrin–radixin-like (MERLIN) tumor suppressor	22q12.2	p.E106* (c.316G>T)	25.02%
			c.363 + 1G>A	2.57%
*EGFR*	Epidermal growth factor receptor	7p11.2	Copy number increase	4.26%
*EPHA2*	EPH receptor A2	1p36.13	p.E241* (c.719dupG)	28.56%
*CDK6*	Cyclin-dependent kinase 6	7q21.2	p.W203* (c.609G>A)	25.7%
*LATS2*	Large tumor suppressor kinase 2	13q12.11	p.Q345* (c.1033C>T)	5.59%
*NBN*	Nibrin	8q21.3	p.E737K (c.2209G>A)	38.64%
*CUL3*	Cullin 3	2q36.2	p.E27K (c.79G>A)	34.76%
*FRAS1*	Fraser extracellular matrix complex subunit 1	4q21.21	p.I2586V (c.7756A>G)	26.79%
*ATM*	ATM serine/threonine kinase	11q22.3	p.D1791N (c.5371G>A)	19.99%
*KMT2A*	Lysine methyltransferase 2A	11q23.3	p.S1425F (c.4274C>T)	19.46%
*EXT1*	Exostosin glycosyltransferase 1	8q24.11	p.Q512H (c.1536G>C)	6.45%
*SMARCA1*	SNF2-related chromatin remodeling ATPase 1	Xq25–q26.1	p.Q28H (c.84G>T)	6.08%
*RECQL4*	RecQ-like helicase 4	8q24.3	p.E1109Q (c.3325G>C)	6.03%
*KMT2D*	Lysine methyltransferase 2D	12q13.12	p.L4462I (c.13384C>A)	5.5%
*POLQ*	DNA polymerase theta	3q13.33	p.A1384V (c.4151C>T)	3.65%
*CTNND2*	Catenin delta 2	5p15.2	p.S436I (c.1307G>T)	0.84%

**Figure 3:**
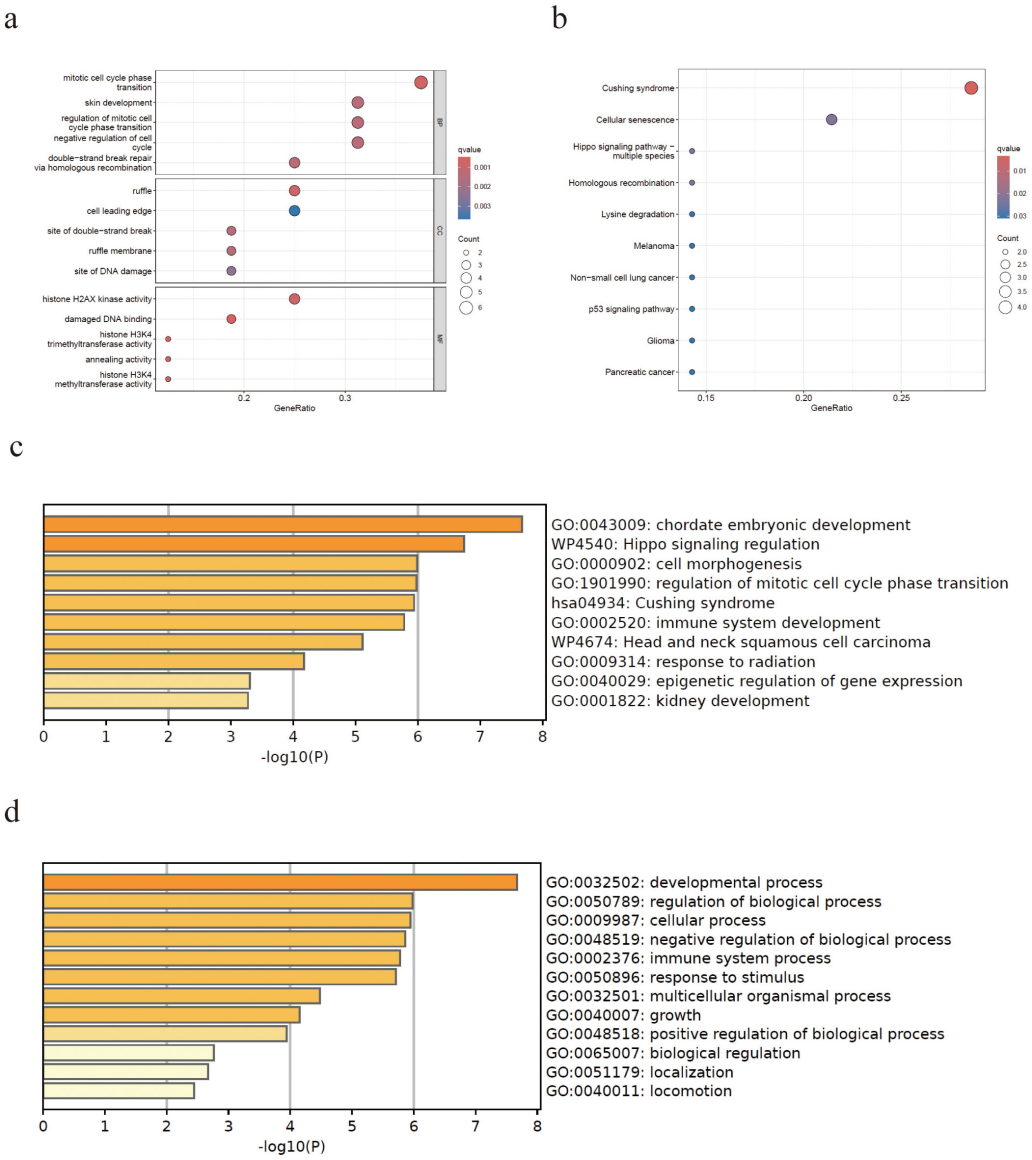
Significantly enriched Gene Ontology (GO) terms and Kyoto Encyclopedia of Genes and Genomes (KEGG) pathways for the mutated genes. **(a)** GO analysis of the biological processes, cellular components, and molecular function. **(b)** KEGG enrichment analysis. **(c, d)** The Metascape database yielded similar results for the mutated genes.

Transcription factors are the key drivers of gene expression and tumorigenesis. Using TRRUST (10) (version 2; https://www.grnpedia.org/trrust/), we mapped the mutated genes to the core transcription factors: *SP1*, *MTA1*, *TP53*, *BRCA1*, *HDAC1*, *AR*, and *RELA* (**Supplementary Table S1**). To uncover their functional roles, we conducted Gene Ontology (GO) and Kyoto Encyclopedia of Genes and Genomes (KEGG) enrichment analyses in Sangerbox 3.0 (http://vip.sangerbox.com). In the GO biological process category, these genes were enriched for mitotic cell cycle phase transition, regulation of mitotic cell cycle phase transition, and negative regulation of the cell cycle (**Figure 3A**). Cellular component analysis placed them predominantly in the ruffle and cell leading edge (**Figure 3A**), while the molecular function terms highlighted H2AX kinase activity and damaged DNA binding (**Figure 3A**). KEGG pathway analysis further linked these factors to Cushing syndrome and cellular senescence (**Figure 3B**), corroborating our Metascape results (**Figures 2C, D**).

Although *TP53* is regarded as the most common mutated gene in GBC, it varies greatly between different studies from 27% to 90% (11–13). Other common mutation genes include *SMAD4*, *ERBB2/3*, *PIK3CA*, and *ARID1A*, among others (11–15). Genomic profiling of 25 patients with GBASC in an Italian study also showed an overlapping prevalence of the driver mutations, including *TP53* (64%), *KRAS* (16%), *CDKN2A* (20%), *PIK3CA* (44%), *PTEN* (24%), and *HNF1A* (12%). Our results demonstrated that the mutational landscape of GBASC shares little features with gallbladder adenocarcinoma. Consequently, we conducted a comparative analysis to further investigate the relationship between these two tumor entities. To investigate the mutation profiles in gallbladder carcinoma, data from the cBioPortal database (https://www.cbioportal.org/) were analyzed using the gallbladder carcinoma dataset [Memorial Sloan Kettering Cancer Center (MSK), Cancer 2018]. As shown in **Supplementary Figure S4A**, *ATM* and *KMT2D* exhibit high mutation frequencies in patients with gallbladder carcinoma. Among 101 sequenced cases, genetic alterations were detected in 16% and 9% of 3,159 gallbladder carcinoma samples, respectively. Furthermore, these genes showed the highest mutation rates in gallbladder carcinoma (33.66% of 101 cases), followed by cholangiocarcinoma (19.66% of 417 cases) and intrahepatic cholangiocarcinoma (16.26% of 412 cases) (**Supplementary Figure S4B**). These results point to a potential critical role of the identified mutations in the development of gallbladder carcinoma.

There are only a few studies that reported on the significant treatment effect of anlotinib on GBASC. However, several surveys have shown that anlotinib combined with chemotherapy or anti-PD-1 antibodies might help control advanced cholangiocarcinoma via blocking the VEGFR2/PI3K/AKT cascade and that it promoted the G0/G1 cell cycle arrest and apoptosis in an *in vitro* experiment (16–18). The bioinformatics analysis of PPI enrichment and the GO and KEGG enrichment of the transcription factors of the 16 mutation genes of this patient mainly focused on cell cycle transition, in particular the G1/S phase. Given that anlotinib has been shown to interfere with proliferative signaling and induce G0/G1 phase arrest, it is plausible that its therapeutic activity in this case is partly attributable to the vulnerability created by these cell cycle-related mutations.

We also searched for studies according to the 16 mutation genes of this patient. An *in vitro* experiment indicated that anlotinib can suppress angiogenesis and resensitize esophageal cancer cells to radiotherapy by inhibiting the expression of *EPHA2* (19). Duan et al. reported on a case of the long-term release of pembrolizumab and anlotinib in thoracic *SMARCA4*-deficient undifferentiated tumor (20). A multicenter, single-arm, prospective phase II trial on sintilimab plus anlotinib for programmed death-ligand 1 (PD-L1)-positive recurrent or metastatic cervical cancer demonstrated that patients with an altered *KMT2D* had a higher objective response rate (ORR) (21). Adversely, in extensive-stage small cell lung cancer, *KMT2D* mutation resulted in shorter progression-free survival (PFS) and overall survival (OS) and higher treatment resistance to sintilimab, anlotinib, and chemotherapy (22). Moreover, tislelizumab and anlotinib have been shown to be effective in a case of *KMT2D*-mutant advanced pancreatic cancer (23).

Furthermore, we explored the relationship between anlotinib and the core transcription factors, including *SP1*, *MTA1*, *TP53*, *BRCA1*, *HDAC1*, *AR*, and *RELA*, and found that anlotinib appears to influence the mutational transcriptional networks that may have contributed to the tumor inhibition in this patient. Mechanistically, it was found that anlotinib could suppress NF-κB/RELA signaling and alter the cell cycle regulation in *TP53*-mutant tumors, resulting in reduced proliferation, stemness, and tumor progression (24, 25). Collectively, there are also studies highlighting that, although anlotinib is not a single-agent direct inhibitor of *BRCA1* or *HDAC1*, it would be efficient when combined with epigenetic drug targeting, indicating the potential bypass role of anlotinib in *BRCA1*- and *HDAC1*-associated pathways (26, 27). Our molecular function terms highlighted H2AX kinase activity and damaged DNA binding. Anlotinib has been shown to increase the H2AX levels in tumor cells, improving hypoxia and enhancing antitumor effects (28).

The controversial content of the treatment of this patient was mainly on the decision for the first operation. According to the 8th edition of the TNM staging system, the stage of this patient was T4N1M0 (IVA) as a locoregionally advanced disease, raising controversy whether extended surgery could achieve longer survival than neoadjuvant systemic therapy. In recent years, several surveys have attempted to confirm that a neoadjuvant approach could play an important role in increasing the radical surgery rate (29–31). The NCCN Guidelines for Gallbladder Cancer (2025) recommend neoadjuvant systemic therapy for locoregionally advanced disease to rule out rapid progression and avoid futile surgery. Fortunately, we achieved R0 resection at the first and the second operation, which would significantly prolong the OS (32). Even with the pathology of neoplasia changing from adenocarcinoma to adenosquamous carcinoma and KI-67 increasing from 60% to 70%, the patient achieved long tumor-free survival with anlotinib combined with an anti-PD-1 antibody as post-surgery adjuvant treatment.

Surgery is recommended for all patients with biliary tract cancer who would achieve R0 resection (33). Furthermore, for metastatic lesions, there are only a few retrospective surveys indicating that the resection of solitary extrahepatic metastases or isolated hepatic recurrences can also deliver prolonged survival and disease-free interval, especially when R0 resection is achievable (34, 35). Secondary R0 resection of the recurrence lesions might have played an important role in this patient’s great prognosis.

Anti-PD-1/PD-L1 antibodies combined with chemotherapy have been recommended as the first-line treatment for advanced biliary tract cancers due to their robust and sustained OS benefit (36, 37). Monotherapy with immune checkpoint inhibitors (ICIs) has also shown modest efficacy in biliary tract cancers (38, 39), when microsatellite instability high or mismatch repair-deficient would help predict therapeutic effects. However, studies have also found that a number of patients with low TMB can still obtain benefits from ICIs (40). The TMB of our patient was low, i.e., 5.73 mut/Mb, sitting at 15% of the gallbladder cancer TMB database. However, she appeared to have benefited from ICIs and target therapy, which could be due to PD-1-positive and some unknown sensitive immune treatment sites in this patient. Furthermore, a low TMB typically indicates lower genomic instability and tumor heterogeneity, which ensure that tumors achieve durable responses to sensitive targeted therapies (41).

This study has some limitations. As a single-case report, this study is inherently limited by its lack of generalizability and the absence of a control or comparison group, which restricts the ability to infer causality or compare outcomes across patient populations. In addition, the statistical interpretations remain preliminary, as a single clinical observation cannot fully delineate the underlying biological pathways. More studies are required to confirm the relationship between the therapy and these mutation genes. Finally, the possibility of a selection or a reporting bias must be acknowledged, as individual cases may not represent the typical clinical course or therapeutic response.

In summary, we report on a rare case of GBASC and describe its clinicopathologic and genomic features. The prognosis is inspiring. Active surgical intervention and the incorporation of gene sequencing into the clinical evaluation may help guide management and improve outcomes. Larger cohorts are needed to identify more effective treatments for GBASC.

## Data Availability

The original contributions presented in the study are included in the article/**Supplementary Material**. Further inquiries can be directed to the corresponding author.

## Supplementary material

Supplementary Figure 1 **(a)** Post-operation biopsy and immunohistochemical analysis of the first surgery revealed poorly differentiated adenocarcinoma with extensive necrosis, positive in PAS, CA199, CK19, CK7, MLH1/2/6, P53 and PMS2 with KI67 for 60%. **(b)** Post-operation biopsy and immunohistochemical analysis of the last surgery revealed poorly differentiated adenosquamous carcinoma, positive in CK19, CK7, MLH1/2/6, MOC31, P53, PMS2, P40 and Vim with KI67 for 70%, PD-1 more than 50%.

Supplementary Figure 2 Timeline graph of this patient’s evolution.

Supplementary Figure 3 The tumor mutation burden quantified by targeted gene sequencing.

Supplementary Figure 4 Gene mutations in gallbladder carcinomas. **(a)** A summary of alterations in the 16 queried gene mutations from the cBioPortal database; **(b)** Detailed summary of alterations in the 16 mutated genes.
