# Supplementary material for: Targeted gene sequencing and bioinformatics analysis of a patient with gallbladder adenosquamous carcinoma: a case report
Source: Front Oncol. 2026 Jan 26;16:1697015. doi: 10.3389/fonc.2026.1697015 (PMC12883791; doi:10.3389/fonc.2026.1697015)
Supplement: Supplementary file 1 [file Table1.docx]

| **Supplementary table 1. Key regulated factor of GBASC related genes** | | | | | |
| --- | --- | --- | --- | --- | --- |
|  | **Key TF** | **Description** | **Overlapped genes** | ***P* value** | **FDR** |
| 1 | SP1 | Sp1 transcription factor | ATM, CDK6, EGFR, RECQL4, SMARCA1 | 3.33E-05 | 2.33E-04 |
| 2 | MTA1 | metastasis associated 1 | EGFR, EPHA2 | 1.84E-04 | 6.44E-04 |
| 3 | TP53 | tumor protein p53 | EGFR, EPHA2, RECQL4 | 3.32E-04 | 7.75E-04 |
| 4 | BRCA1 | breast cancer 1, early onset | ATM, EGFR | 1.05E-03 | 1.83E-03 |
| 5 | HDAC1 | histone deacetylase 1 | EGFR, RECQL4 | 1.62E-03 | 2.27E-03 |
| 6 | AR | androgen receptor | CDK6, EGFR | 2.76E-03 | 3.22E-03 |
| 7 | RELA | v-rel reticuloendotheliosis viral oncogene homolog A (avian) | CDK6, EGFR | 2.63E-02 | 2.63E-02 |
