# Supplementary figures and images for: Targeted gene sequencing and bioinformatics analysis of a patient with gallbladder adenosquamous carcinoma: a case report
Source: Front Oncol. 2026 Jan 26;16:1697015. doi: 10.3389/fonc.2026.1697015 (PMC12883791; doi:10.3389/fonc.2026.1697015)

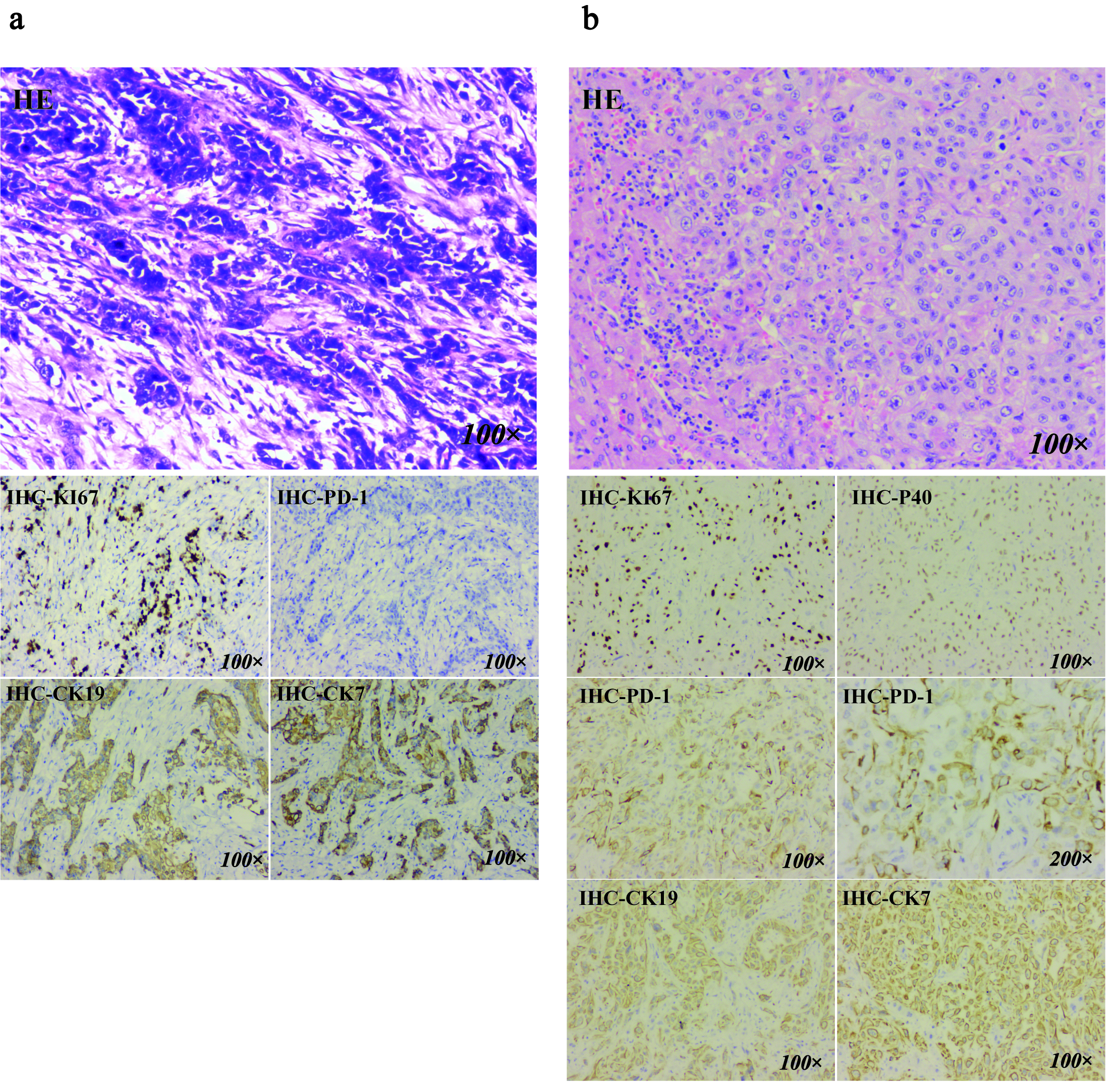

Supplement: Supplementary Figure 1 — (a) Post-operation biopsy and immunohistochemical analysis of the first surgery revealed poorly differentiated adenocarcinoma with extensive necrosis, positive in PAS, CA199, CK19, CK7, MLH1/2/6, P53 and PMS2 with KI67 for 60%. (b) Post-operation biopsy and immunohistochemical analysis of the last surgery revealed poorly differentiated adenosquamous carcinoma, positive in CK19, CK7, MLH1/2/6, MOC31, P53, PMS2, P40 and Vim with KI67 for 70%, PD-1 more than 50%. [file Image1.tif]

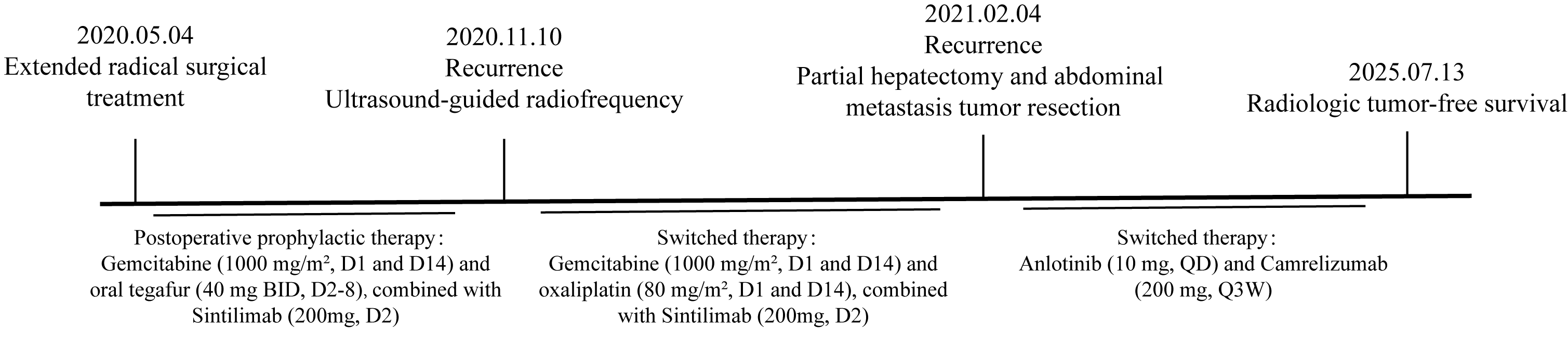

Supplement: Supplementary Figure 2 — Timeline graph of this patient’s evolution. [file Image2.tif]

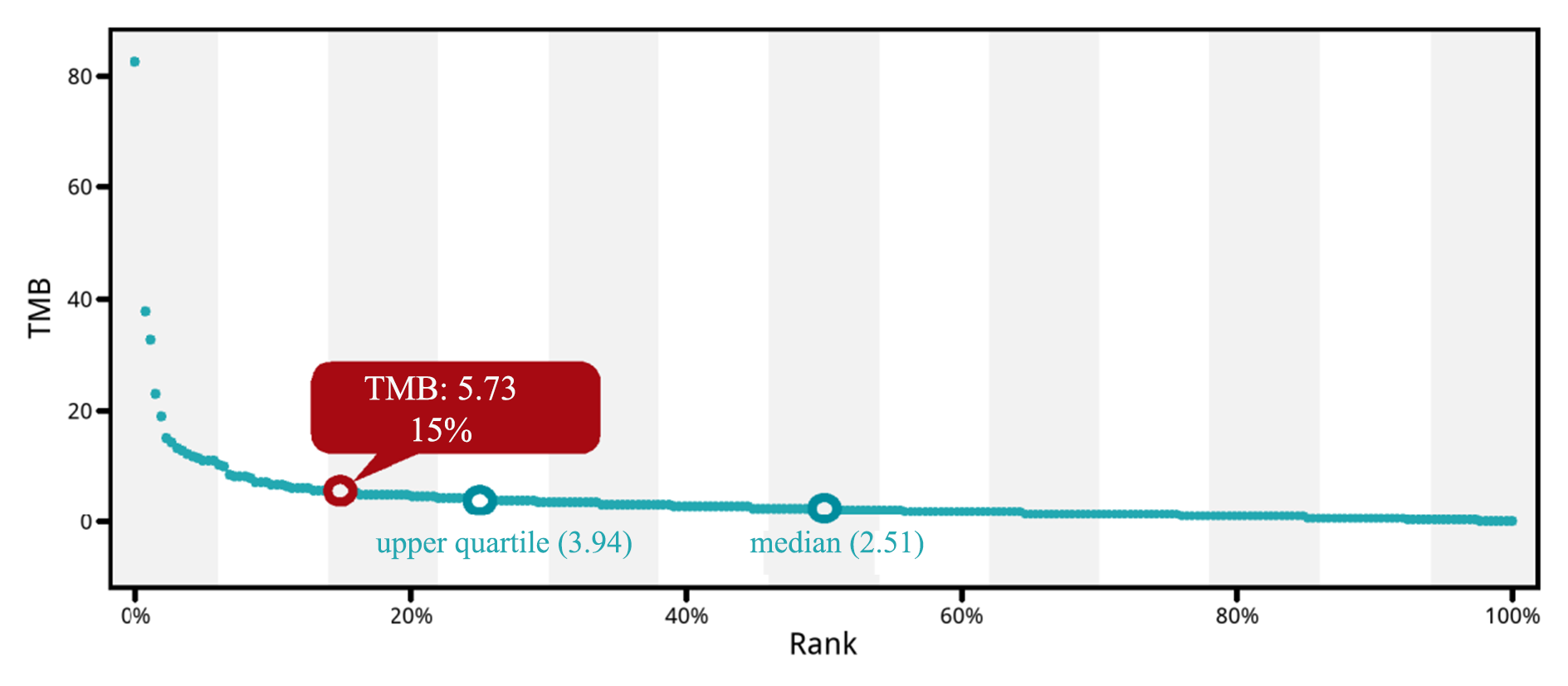

Supplement: Supplementary Figure 3 — The tumor mutation burden quantified by targeted gene sequencing. [file Image3.tif]

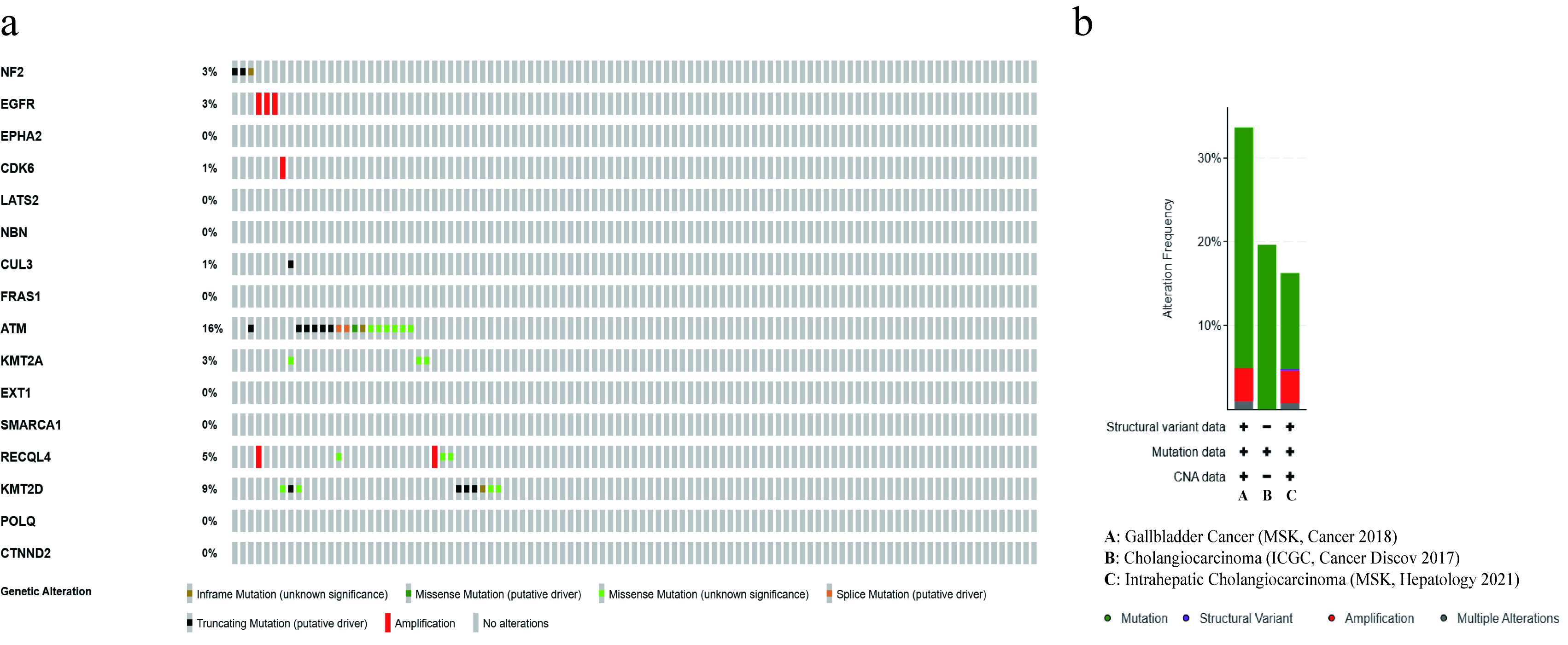

Supplement: Supplementary Figure 4 — Gene mutations in gallbladder carcinomas. (a) A summary of alterations in the 16 queried gene mutations from the cBioPortal database; (b) Detailed summary of alterations in the 16 mutated genes. [file Image4.tif]
